# Microscopic characterization of longitudinal heat-induced fractures in calcined human remains

**DOI:** 10.1007/s00414-026-03869-z

**Published:** 2026-06-16

**Authors:** Debora Mazzarelli, Annalisa D’Apuzzo, Giulia Caccia, Riccardo Solazzo, Lorenzo Franceschetti, Noelle Maggiori, Cristina Cattaneo, Annalisa Cappella

**Affiliations:** 1 LABANOF, Laboratory of Forensic Anthropology and Odontology, Institute of Legal Medicine, Department of Biomedical Sciences for Health, University of Milan, Milan, Italy; 2 Department of Biomedical Sciences for Health, University of Milan, Milan, Italy; 3 U.O. Laboratorio di Morfologia Umana Applicata, IRCCS Policlinico San Donato, San Donato Milanese, 20097 Italy

**Keywords:** Burnt bones, Fractures, Bone, Microscopic analysis, Osteons, Harvesian System, Heat-induced osteonal models.

## Abstract

Fire is a primary method of corpse destruction, complicating victim identification and the determination of cause and manner of death. This study focuses on the microscopic examination of fracture behavior in osteonal structures, referred to as osteonal damage analysis, to distinguish heat-induced fractures from perimortem and postmortem mechanical fractures. Thin sections of calcined bone were compared with perimortem and postmortem blunt trauma samples to investigate differences in the fracture behavior of osteonal structures. Statistical analysis using a Generalized Linear Mixed Model (GLMM) revealed significant variations (*p* < 0.05) in fracture patterns. Fractures predominantly crossed osteons, either cutting through the Haversian canal or following the cement line. While significant differences in fracture profiles were found, no pattern was exclusive to any group, with all fracture patterns present across all groups in varying frequencies. Crucially, the findings related to the calcined group suggest, for the first time, that heat-induced longitudinal fractures can intersect the Haversian canals, challenging the longstanding belief that only transverse fractures cross osteons transversely. These findings underscore the complexity of differentiating heat-induced from mechanical fractures and highlight the need for further research. A deeper understanding of osteonal damage is crucial for advancing forensic analysis of burned remains.

## Introduction

Human remains can be exposed to fire under various circumstances, including funerary rituals, vehicle or aircraft accidents, natural disasters, homicides, or suicides [1, 2]. In confined environments like cars or small apartments, temperatures exceeding 1000 °C, comparable to cremation ovens [3, 4], can lead to nearly complete body destruction, resulting in calcination and severe bone fragmentation. Factors such as obesity, flammable clothing [3] and accelerants [5] can even amplify fire damage. However, complete combustion is rare with differential anatomical preservation being more common [3]. In the remains, thermal damage can mask other perimortem traumas, such as mechanical lesions. On the other hand, heat-induced lesions may potentially mimic other forms of injury and be misclassified as perimortem mechanical lesions by forensic pathologists and anthropologists [6]. Overall, distinguishing heat-induced fractures from other traumas is crucial for understanding the manner and cause of death of the victim [7, 8]. The scientific community has therefore been developing methods to differentiate heat-induced fractures from peri-mortem [9] and post-mortem mechanical fractures [10]. To date, research has focused on the effects of heat on peri-mortem injuries in soft tissues and skeletal remains, identifying differences in the extent of peri-mortem fractures, patterns, and lesion margins through macroscopic analysis [10, 11]. Despite these observations, mechanical fractures in cremated bones are often difficult to identify and become indiscernible in approximately 70% of cases [11, 12]. At the same time, fractures lacking heat-related features are often interpreted as thermal fractures based only on the forensic expert’s subjective experience [11]. Several studies have highlighted the challenges of identifying traumatic injuries in burned remains and linking them to associated weapon types, emphasizing the need for a deeper understanding of heat-induced and mechanical fractures [13–17]. However, recognition of fracture type based solely on macroscopic features remains challenging [18]. From this standpoint, microscopic analysis of fracture margins using optical microscopy has already been applied to study bone trauma with the intent to improve the challenging interpretation of skeletal lesions [19–21]. Pechníková et al. [20], through a microscopic evaluation of fracture propagation models in fresh and dry human bones, revealed that fractures tended to cross Haversian canals or osteon lamellae, while only in a minor percentage (about 35%) the fracture line propagated around the internal lamellae or the cement line regardless the bone typology (whether fresh or dry). In another study based on Piekarski’s theory [22], which states that fractures propagation depends on the velocity of the trauma to which the bone is exposed, Pechníková et al. [18] microscopically evaluated adult pig bones subjected to low-velocity blunt force trauma due to manual compression with a vice and a car run-over and high-velocity trauma caused by an explosion. The authors found a statistically significant difference in the fracture behavior between the two types of injury: in 74% of high-velocity trauma, the fracture crossed the Haversian canal, whereas in only 45% of low-velocity blunt trauma the fracture behaves the same. However, in both studies [18, 20] the authors concluded that no specific fracture pattern could be conclusively linked to a specific injury type. Interestingly, a study conducted by Sexton et al. [23] supports the hypothesis that distinct patterns of osteonal damage exist within human long bones subjected to blunt force and gunshot trauma, suggesting that fractures crossing the Haversian canal may be indicative of blunt force injury. Gunshot fractures were also evaluated by Kieser et al. [24] who, using pig ribs subjected to high-velocity trauma from a gunshot at close range (5 cm), described that fractures follow the fatty deposit within the cortical bone at the entrance of the bullet, then cross osteons rather than passing around them until the exit of the bullet. Additionally, to blunt force and gunshot trauma, microscopic analysis was applied also to other types of traumas and fractures. Research on sharp force trauma produced on human humeral diaphysis ten years post-autopsy demonstrated that fracture lines are more common in single-stroke injuries, radiating from the kerf floor due to the combined impact of cutting and blunt forces [21]. The study also demonstrated that osteons are severed by fracture lines, although lamellar structures may act as barriers to secondary fracture propagation [21] the full understanding of which fracture patterns are associated with a particular types of bone trauma remains challenging.

Overall, microscopic analysis has also been applied to burned remains, where the microscopic osteological structure, although modified, often remains intact, unlike biomolecular components such as proteins and DNA, which degrade at high temperatures [25, 26]. Pioneering studies on cremated bones revealed irregular-width cracks along the Haversian system’s periphery, connecting with other fissures under heat exposure [19].

Later research identified fractures radiating inward from the bone surface, transitioning from calcined to charred areas, with lamellar fractures surrounding Haversian canals in calcined bone [27], while Hanson et Cain (2007) observed that cracks in sheep bones began near Haversian canals at around 480 °C and extended to bone edges above 700 °C [28]. Discrepancies in the literature also exist regarding dimensional changes in bone microstructures during combustion and particularly concerning osteon size, with studies reporting either a decrease [19, 25, 26, 29, 30] or an increase [31, 32] with rising temperatures.

Given the value of the osteonal damage patterns analysis in forensic anthropology [23] and the existing literature gaps (specifically, the lack of a detailed analysis of the macroscopic and microscopic presentation of fractures produced by different types of trauma), this study aims to investigate the microscopic characteristics of heat-induced lesions in human cortical bone, focusing on longitudinal fractures, the most commonly observed fracture type in case of burned human long bones [33]. In particular, the primary aim is to identify microscopic fracture propagation patterns that characterize heat-induced longitudinal fractures, which may potentially help to differentiate these lesions from others such as perimortem and postmortem mechanical injuries. To achieve this, comparative observations were made on longitudinal fractures from calcined human remains (produced by heat), and from fresh autopsy samples and dry bones dated to the 16th century (produced by blunt force trauma). Intra-observer repeatability and inter-observer reproducibility were also tested to ensure the reliability of the findings.

## Materials and methods

### Materials

The sample used in this study consists of unclaimed calcined bone fragments obtained from a crematorium and included in the LABANOF Anthropological Collection (CAL) of the Laboratory of Forensic Anthropology and Odontology (LABANOF) at the University of Milan. These remains were deemed suitable for research in accordance with Article 43 of the Italian Presidential Decree (D.P.R.) No. 285 of 10 September 1990, which regulates mortuary police activities. It is important to note that modern crematoria in Italy are gas-fueled, and the incineration temperature generally ranges between 870 °C and 980 °C, with an average process duration of 2 to 2.5 h (although this may vary depending on body mass).

The calcined bone fragments were selected according to the following criteria: (i) state of preservation allowing the gross morphological identification of the bone or, more generally, the anatomical district of origin (e.g., upper or lower extremities); (ii) presence of longitudinal fractures related to heat exposure [34]; (iii) long bone diaphyseal origin, to ensure thicker cortical bone and, consequently, a potentially greater number of osteons to observe. The selected 19 bone fragments were cut transversely to the long axis of the bone using a saw to obtain more analyzable specimens, and a total of 49 thin sections were prepared and analyzed. The thin sections obtained from the calcined fragments, designated as Group 1, were then compared with control thin sections deriving from a previous study [20], which included both perimortem and postmortem longitudinal fractures on fresh and dry bones. In this study, a subsample of the thin sections originally analyzed by Pechnikova et al. [20] was evaluated: the perimortem fractures sample (Group 2) included 19 thin sections obtained from diaphyseal fragments with longitudinal fractures caused by blunt force trauma collected during autopsies; the postmortem fractures sample (Group 3) consisted of 31 thin sections retrieved from diaphysis of 16th -century skeletons experimentally fractured (from LABANOF Anthropological Collection (CAL) [35]. Indeed, the total sample included 99 thin sections.

### Preparation of thin sections

The thin sections were prepared following a standard protocol commonly used for histological investigations on undecalcified bone tissue [26, 36, 37]. Each bone fragment was labeled and transversely sectioned using a hacksaw into multiple portions measuring between 1 and 2 cm to analyze various points of the same fracture. Due to the high fragility of the calcined samples, a consolidation phase of 24–48 h was carried out using Araldite mixed with a hardener at a ratio of 1.5 g of hardener for every 10 g of resin. Subsequently, each section underwent grinding with a Struers Dap-7 geological grinder, using abrasives with progressively finer grit (from 180 to 1200). After this stage, the ground specimens were embedded in Araldite again, a necessary step that, although slowed down the procedure, prevented the formation of further cracks and fractures within the sample due to the insufficient penetration of the Araldite into the damaged bone tissue. A second grinding and polishing process was then carried out. The polished surface of each specimen was mounted on a glass slide using Eukitt^®^ mounting medium and left to dry for at least 24 h. Subsequently, the other side was ground until a thickness of approximately 100 μm was reached, monitored under an optical microscope to avoid material loss. Finally, the obtained thin slices were polished using abrasives with grits of 2400 and 4000 to remove any scratches caused by the previous stages. Histological analysis was performed using an Axio Scope.A1^®^ optical microscope connected to a Tucsen’s TrueChrome II HD^®^ camera. Observations were made at magnifications of 100x and 200x, and the photographs were captured using the IScapture^®^ software.

### Microscopic observation of the morphological pattern

**Fig. 1:**
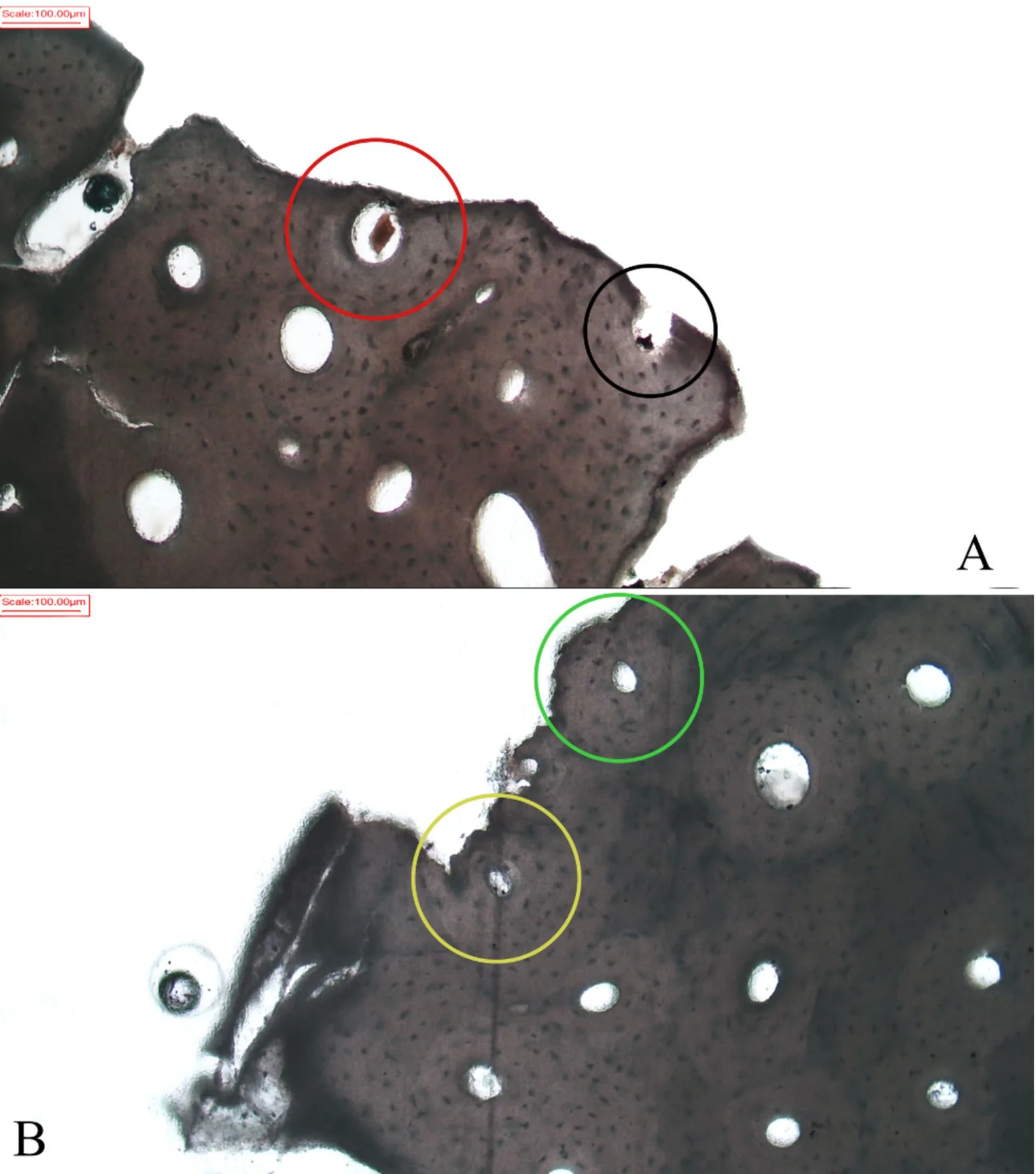
(**A**-**B**) thin sections of calcined bone (x100 magnification). Circles highlight the osteon residing at the fracture margin: Black indicates Pattern 1; Red indicates Pattern 2; Green indicates Pattern 3; and Yellow indicates Pattern 5

The analysis involved the observation of the margins of the longitudinal fracture in a cross-sectional view to study how the fracture line progresses through the bone tissue in relation to the secondary osteonal structures. For the evaluation, five reference fracture Patterns were defined: the first four were formulated based on previous descriptions [20], while the fifth model was derived from preliminary observations of thin sections from Group 1. Table 1 provides descriptions and graphical representations of each pattern type used for the evaluations, also represented in Fig. 1 in a real thin section of a calcined fragment.

**Table 1:**
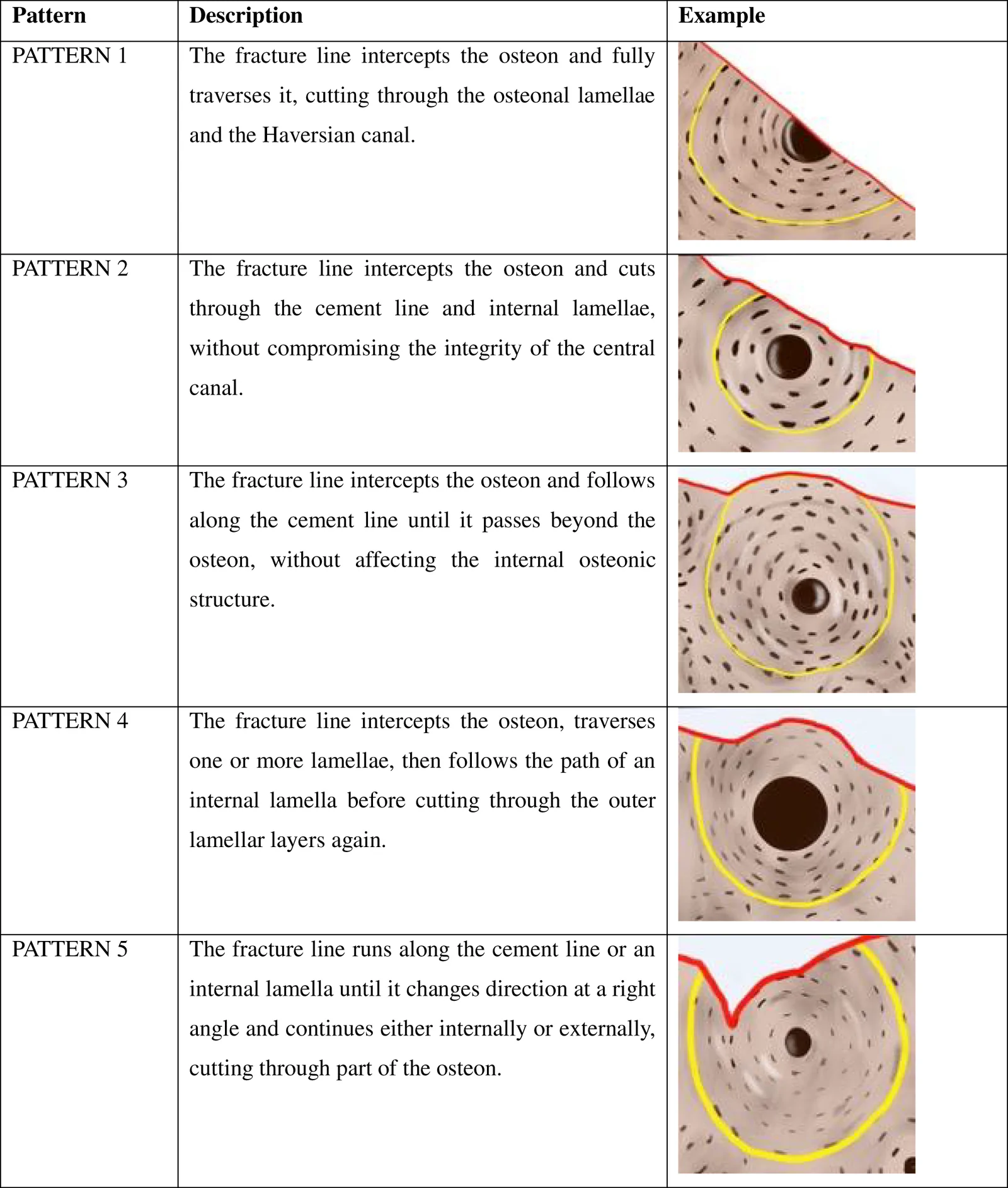
Reference fracture patterns used in the study. The red line indicates the fracture line; the yellow line indicates the cement line of the osteon

### Intra- and inter-operator reliability

Following an initial evaluation conducted by one trained operator (Operator 1) on the calcined slides and the perimortem and postmortem lesions, twenty sections (ten from Group 1, five from Group 2, and five from Group 3), representing approximately 20% of the total sample, were selected to verify the repeatability of the observations. One month after the initial evaluation, the same slides were re-evaluated by Operator 1 (intra-operator repeatability) and by a second expert operator (inter-operator reproducibility) with an experience of 10 years in microscopic analysis.

### Statistical analysis

The intra- and inter-operator reliability were assessed using the intraclass correlation coefficient (ICC). ICC values and the corresponding 95% confidence interval (CI) were calculated based on a single measurement and absolute agreement and interpreted according to Koo and Li [38]. For each group and osteon pattern, an intraclass correlation coefficient was obtained ranging from 0 to 1 with values below 0.5 indicating poor repeatability, values between 0.5 and 0.75 moderate repeatability, values between 0.75 and 0.9 good repeatability, and values above 0.9 excellent repeatability.

The total number of osteons per group and the average number of osteons per thin section in each group were calculated. Additionally, the average number of osteons per pattern in each group was then evaluated to ascertain the trend (frequency) of the different patterns in each lesion group.

A Generalized Linear Mixed Model (GLMM) was used to examine the effects of Group, Pattern and their interaction (fixed factors) on the number of osteons, while accounting for variability of the thin sections (random factor). The model used a Poisson distribution with logarithmic link function, a framework commonly used for count data. As the fixed factors had more than two levels, in case of significant effects, post-hoc pairwise comparisons with a step-down Bonferroni correction were performed. Initially, the comparisons were made between Group 1 (heat-induced fractures), Group 2 (perimortem fractures) and Group 3 (postmortem fractures) to assess statistically significant differences in terms of mean number of osteons. The same analysis was also used to compare the number of osteons for each Pattern observed within each group’s samples, and so to identify any trends within groups or differences between groups. Furthermore, a chi-square (χ^2^) analysis of independence was performed to examine the possible relationship between the Groups and the Patterns. The significance was set at α = 0.05 for all analysis, which were performed in SPSS version 29.0.2 (IBM Corp., Armonk, NY, USA).

## Results

The intra- and inter-operator reliability proved to be outstanding in all the different examined groups regardless of the pattern, with ICC values always greater than 0.945. Considering each pattern, the intra-operator repeatability was consistently excellent with all the intraclass correlation coefficient (ICCs) values greater than 0.934. The same was found for the inter-operator reproducibility for the microscopic Patterns 1, 2 and 5, which proved excellent ICC values (above 0.956), while the ICCs of Patterns 3 and 4, although almost excellent, showed slightly lower values (0.893 and 0.865, respectively). Table 2 presents the intra- and inter-operator reliability of the microscopic evaluation for each group and osteon pattern. Along with the ICC values, the extremes of the 95% confidence interval (CI) are reported.

**Table 2:** ICC values for intra- and inter-operator reliability are reported for each group and osteon pattern, along with the extremes of the 95% confidence interval (CI)

	Intra-operator repeatability		Inter-operator reproducibility	
	ICC	95% CI	ICC	95% CI
Group				
1	0.990	0.984–0.993	0.976	0.963–0.984
2	0.993	0.988–0.996	0.971	0.950–0.983
3	0.977	0.960–0.987	0.945	0.901–0.969
Pattern				
1	0.993	0.986–0.996	0.983	0.967–0.991
2	0.985	0.972–0.992	0.956	0.918–0.977
3	0.934	0.877–0.966	0.865	0.757–0.927
4	1.000	/	0.893	0.802–0.943
5	1.000	/	1.000	/

A total of 1364 osteons were examined across the entire sample of 99 thin sections. In Group 1 (calcined samples), 594 osteons were identified across 49 thin sections. For what concern Group 2 (experimentally induced longitudinal perimortem blunt lesions), 399 osteons were analyzed from 19 sections, while in Group 3 (experimentally induced longitudinal postmortem blunt fractures) 371 osteons were observed in 31 sections. In Table 3, the data regarding the average number of osteons observed per slide are reported. This value represents the mean number of osteons in each thin section for each group under analysis, which proved similar for Group 1 and Group 3. All osteons examined in the three groups were classified according to one of the five patterns, and all five descriptive patterns were observed in each group, although with varying frequencies.

**Table 3:** Data on the average number of osteons and the predominant osteon pattern observed per slide are presented

Group	Number of thin sections	Total number of osteons	Average osteon number per thin section	Average osteon number per pattern				
				1	2	3	4	5
1	49	594	12.1	7.2	1.7	3.0	0.1	0.1
2	19	399	21.0	7.8	4.3	7.5	1.0	0.4
3	31	371	12.0	5.4	1.8	4.2	0.3	0.3
Total	99	1364	/	/	/	/	/	/

**Fig. 2:**
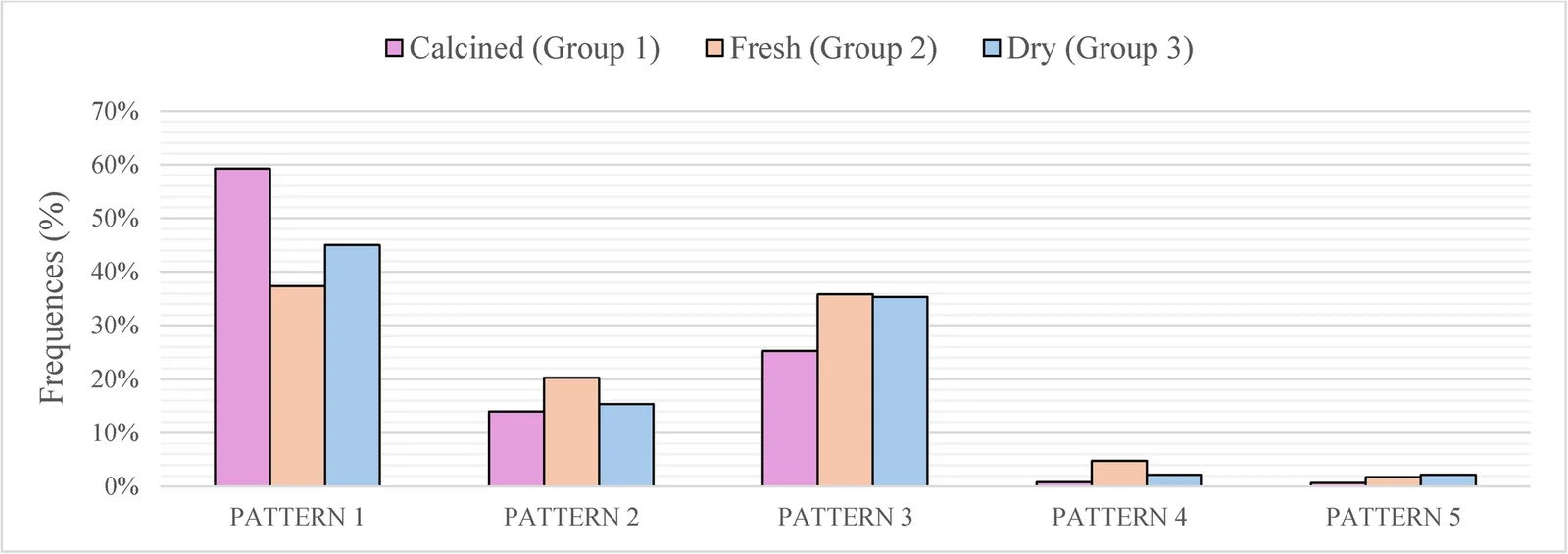
Histogram of the percentages of osteons present for each pattern out of the total osteons analyzed in the three sample groups considered. Data for Group 1 are in pink, those for Group 2 are in orange, while those for Group 3 are in light blue

The percentages of osteons observed for each Pattern relative to the total number of osteons considered for that pattern, as illustrated in Fig. 2, indicate that Pattern 1 and Pattern 3 are the most frequent patterns in Group 1, representing approximately 60% and 25%, respectively, of the total number of osteons observed for this group. Similarly, in Group 2 the highest percentages correspond to Pattern 1 and Pattern 3, which together account for more than 70% of the analyzed osteons (37% for Pattern 1 and 36% for Pattern 3). In Group 2, Pattern 2 is detected in a significant proportion, amounting to 20%. Finally, in Group 3, Pattern 1 dominates with 45% of observed osteons, followed by a slightly lower percentage for Pattern 3 (35%), while Pattern 2 is present in 15% of the cases. Patterns 4 and 5 are observed to a lesser extent in all groups, with percentages consistently below 5% of the total.

The Generalized Linear Mixed Model (GLMM) results comparing the three Groups, the different Patterns, and their interaction (Group*Pattern) are shown in Table 4.

**Table 4:** Two-way ANOVA test results with relevant p-values

Variable	F-Value	*p*-Value
Group	13.952	< 0.001^§^
Pattern	125.566	< 0.001^§^
Group*Pattern	7.014	< 0.001^§^

^**§**^indicates significant differences at α = 0.05

The post-hoc comparisons for the Group variable showed that Group 2 is significantly different than Groups 1 (*p* < 0.001) and 3 (*p* = 0.003), as it is characterized by a greater number of osteons regardless of the pattern. Contrarily, no differences were observed between Group 1 and Group 3 (*p* = 0.108). The pairwise comparisons of the pattern analysis, regardless of the group, revealed that all Patterns differ between each other (all *p* < 0.001), except for Pattern 4 and 5 (*p* = 0.697) as characterized by a similar number of osteons. The comparative analysis of groups and fracture patterns is summarized in Table 5. Pattern 1 did not show any statistically significant differences among the groups, although it was less frequently observed in Group 3. In contrast, Pattern 2 was significantly more prevalent in Group 2 compared to Group 1 (*p* = 0.001) and Group 3 (*p* = 0.022), while no significant difference was found between Group 1 and Group 3 (*p* = 1.000). Similarly, Pattern 3 occurred significantly more frequently in Group 2 than in Group 1 (*p* < 0.001) and Group 3 (*p* = 0.016). Regarding Pattern 4, characterized by a fracture line that intersects one or more osteon lamellae and then follows its path, a significant difference was found between Group 1 and Group 2 (*p* < 0.001), whereas no significant differences were observed between Group 1 and Group 3 (*p* = 0.958) or between Group 2 and Group 3 (*p* = 0.108). Finally, for Pattern 5, in which the fracture line runs along the cement line or an internal lamella and subsequently changes direction to continue either within or outside the osteon, no statistically significant differences were detected among the three groups.

**Table 5:** Post-hoc comparisons between groups for each pattern considered

	Group comparison	*p*-Value
Pattern 1	1 vs. 2	0.998
	1 vs. 3	0.991
	2 vs. 3	0.703
Pattern 2	1 vs. 2	0.001*
	1 vs. 3	1.000
	2 vs. 3	0.022*
Pattern 3	1 vs. 2	< 0.001*
	1 vs. 3	0.854
	2 vs. 3	0.016*
Pattern 4	1 vs. 2	< 0.001*
	1 vs. 3	0.958
	2 vs. 3	0.108
Pattern 5	1 vs. 2	0.448
	1 vs. 3	0.876
	2 vs. 3	1.000

* indicates significant differences at α = 0.05

The results of the within-group comparisons are presented in Table 6. This analysis assessed statistically significant differences in the number of osteons associated with each fracture pattern within the same group. In Group 1, all pairwise comparisons were statistically significant except for the comparison between Pattern 4 and Pattern 5 (*p* = 1.000). Groups 2 and 3 exhibited similar trends, as no significant differences were observed between Pattern 1 and Pattern 3 (*p* = 1.000 and *p* = 0.748, respectively), and between Pattern 4 and Pattern 5 (*p* = 0.622 and *p* = 1.000, respectively), while all remaining comparisons yielded statistically significant differences.

**Table 6:** Post-hoc comparisons (p-value) of Patterns within each Group

Pattern Comparison	Group		
	Group 1 (calcined)	Group 2 (fresh)	Group 3 (Dry)
1 vs. 2	< 0.001*	0.001*	< 0.001*
1 vs. 3	< 0.001*	1.000	0.748
1 vs. 4	< 0.001*	< 0.001*	< 0.001*
1 vs. 5	< 0.001*	< 0.001*	< 0.001*
2 vs. 3	0.006*	0.005*	< 0.001*
2 vs. 4	< 0.001*	< 0.001*	< 0.001*
2 vs. 5	< 0.001*	< 0.001*	< 0.001*
3 vs. 4	< 0.001*	< 0.001*	< 0.001*
3 vs. 5	< 0.001*	< 0.001*	< 0.001*
4 vs. 5	1.000	0.622	1.000

* indicates significant differences at α = 0.05

A chi-square (χ²) test of independence was conducted to assess the relationship between fracture patterns and groups, revealing a statistically significant association between the two variables (χ² = 62.54, *p* < 0.001). To further explore this association, standardized residuals were calculated for each cell. According to the commonly accepted threshold, residuals with absolute values greater than 2 were considered meaningful contributors to the observed association. A positive standardized residual indicates that a given pattern is overrepresented in a group relative to the expected frequency, while a negative residual indicates underrepresentation Table 7.

**Table 7:** Chi-square analysis

		Pattern1	Pattern2	Pattern3	Pattern4	Pattern5
Group 1	Observed	352	83	150	5	4
	Expected	290.9	96.2	184.6	13.9	8.3
	Standardized residual	3.6	-1.3	-2.5	-2.4	-1.5
Group 2	Observed	149	81	143	19	7
	Expected	195.4	64.6	124.0	9.4	5.6
	Standardized residual	-3.3	2.0	1.7	3.2	0.6
Group 3	Observed	167	57	131	8	8
	Expected	181.7	60.1	115.3	8.7	5.2
	Standardized residual	-1.1	-0.4	1.5	-0.2	1.2

Overall, Group 1 (Calcined sample) and Group 2 (Fresh sample) exhibited the largest standardized residuals, indicating that their pattern distributions deviated significantly from what would be expected under a random association. Specifically, the Calcined sample showed an overrepresentation of Pattern 1 (standardized residual: +3.6) and an underrepresentation of Patterns 3 and 4 (residuals: -2.5 and − 2.4, respectively). In contrast, the Fresh sample was characterized by a lower frequency of Pattern 1 (residual: -3.3) and an overrepresentation of Patterns 2 (+ 2.0) and 4 (+ 3.2). Group 3 (Dry sample) did not display any residuals exceeding ± 2, suggesting that its Pattern distribution aligns with expected frequencies under the null hypothesis.

## Discussion

Due to its destructive effects, fire is a commonly employed method for body concealment, as it reduces the chances of identification of victims and interpretation of the manner and cause of death [39–42]. Understanding whether a body was burned before burial, shortly after, or long after burial, and whether it shows other signs of trauma, is of primary importance for forensic experts [4]. Forensic research has made substantial progress in the characterization of bone fractures. Indeed, several studies have investigated the morphological features of fractures in relation to their causative mechanisms [2, 10–18, 21–24, 27, 43–50] as well as their timing and healing stage [6, 20, 51–57] through either microscopic, macroscopic, or radiological approaches. Indeed, the sole observation of macroscopic and radiological features seem to help the interpretation of trauma and fracture mechanism formation when evaluated within the context of known circumstances [18]. However, most of these studies have been performed on well-preserved or experimentally prepared bone samples, either fresh or dry. Consequently, the identification and interpretation of trauma in taphonomically altered bones remained largely unexplored. This gap is particularly evident for thermally altered, fragile remains, where heat damage may mask perimortem traumas or simultaneously mimic mechanical traumas, leading to misinterpretation by forensic pathologists and anthropologists [43, 47, 48, 50]. The differentiation between heat- and trauma-related fractures is further complicated by the fact that thermal alterations are often more intense at pre-existing trauma sites than in intact areas [58].

The current forensic literature lacks in-depth analysis of trauma in burned human bones, and the available studies primarily focus on macroscopic observation [10, 11, 14] or microscopic structural changes following burning [19, 27, 28]. Conversely, studies on microscopic features and histological appearance of skeletal trauma in unburned bones are largely focused on the characterization of mechanical injuries [20, 21, 23, 24] peri- and post-mortem timing of the fractures [6, 20, 51–57] and the estimation of the post-traumatic interval [52, 59], while no studies investigating osteonal damage characteristics of heat-related fractures have been conducted in burned remains. The combination of prolonged burning times, high temperatures, and the presence of dry tissue can lead to massive skeletal destruction, with minimal bone recovery [4]. In such cases, much evidence of trauma may be lost with the loss of cortical bone due to heat [25, 58, 60]. This appears to be true both for cutting injuries, regardless of the type of blade used, as demonstrated in a study on pig ribs [60], and for blunt force trauma, as shown in studies on human cranial and pelvic bones [11, 58]. However, even when only small bone fragments are recovered, such as in cremation, the histological structure can still be intact and available for observation, even in bones subjected to long periods of taphonomic exposure [4, 23]. In a study on the variation of the cortical bone of human ribs during cremation, the bone microstructure was evident at all cremation temperatures used, up to 1000 °C [25]: although the microstructures in the burnt bones were difficult to identify and distinguish from one another, intact osteons and Haversian canals could be still reliably identified. But apart from investigations on the preservation of bone microstructure, no study has explored the behavior of heat-induced fractures in relation to the osteon structure in the cortical bone yet. This research investigated the microscopic characteristics of heat-induced longitudinal fractures in calcined human cortical bones, comparing them with longitudinal blunt force/mechanical fractures in fresh and dry bone samples (perimortem and postmortem longitudinal fractures, respectively). This type of fracture appears in human cortical and trabecular bone exposed to heat starting at temperatures of 100–200 °C, becoming more evident between 300 °C and 400 °C [61].

In this study, five distinct microscopic fracture patterns were defined based on the behavior of fracture lines in relation to osteons at the fracture margins, with modifications to the classification initially proposed by Pechnikova et al. [20]. Whereas their analysis identified only two patterns—Pattern A (fracture lines crossing the Haversian canal or lamellae) and Pattern B (fracture lines following the cement line or an inner lamella)—we redefined the four scenarios as separate characteristics (Patterns 1–4). Additionally, a fifth pattern, exhibiting a mixed trajectory, was identified following preliminary examination of the calcined bone sections. This pattern is characterized by a fracture line initially following a cement line or inner lamella before crossing the osteon. According to our descriptive fracture patterns, a total of 1364 osteons were evaluated within the 99 thin sections obtained from the three bone groups: calcined, fresh, and dry. The number of thin sections evaluated varied across the groups, with the calcined samples being the most represented in order to obtain a sufficient number of osteons for analysis, due to the challenging observability of these specimens. The preparation of these samples was time-consuming due to the repeated steps of embedding in Araldite, further complicated by the small and fragile nature of the bone fragments. Indeed, bone becomes extremely fragile after exposure to high temperatures (close to 1000 °C) for prolonged periods, leading to the loss of water and organic components. This makes the sectioning and grinding process more challenging. Additionally, the interpretation of perimortem and postmortem fractures (fresh and dry bone tissue) was easier compared to the fractures of the calcined tissue where the reduced light transmission is likely due to hydroxyapatite crystal growth, increased crystallinity, and decreased porosity as temperature rises [62], further complicating interpretation. However, excellent intra- and inter-operator reliability (> 0.90) was proved for all groups and for almost all patterns regardless of the state of preservation of the tissue characterizing the Group and the experience of the operator.

The Generalized Linear Mixed Model (GLMM) revealed significant inter-group differences for Patterns 2, 3, and 4. Group 2 (perimortem) showed a higher average number of osteons affected by fractures crossing osteonal lamellae (Pattern 2) and following either the cement line (Pattern 3) or an inner lamella (Pattern 4), compared to Groups 1 (calcined) and 3 (postmortem). No differences were observed for Patterns 1 and 5, although Pattern 1 was more recognizable in calcined and perimortem samples. In Group 1, fractures crossing the Haversian canal (Pattern 1) dominated (> 59%), while Patterns 4 and 5 were rare across all groups. Both fresh and dry bones shared similar frequencies of Patterns 1 and 3, supporting prior findings that fracture progression is more influenced by impact velocity than bone condition as resumed in Table 8.

**Table 8:**
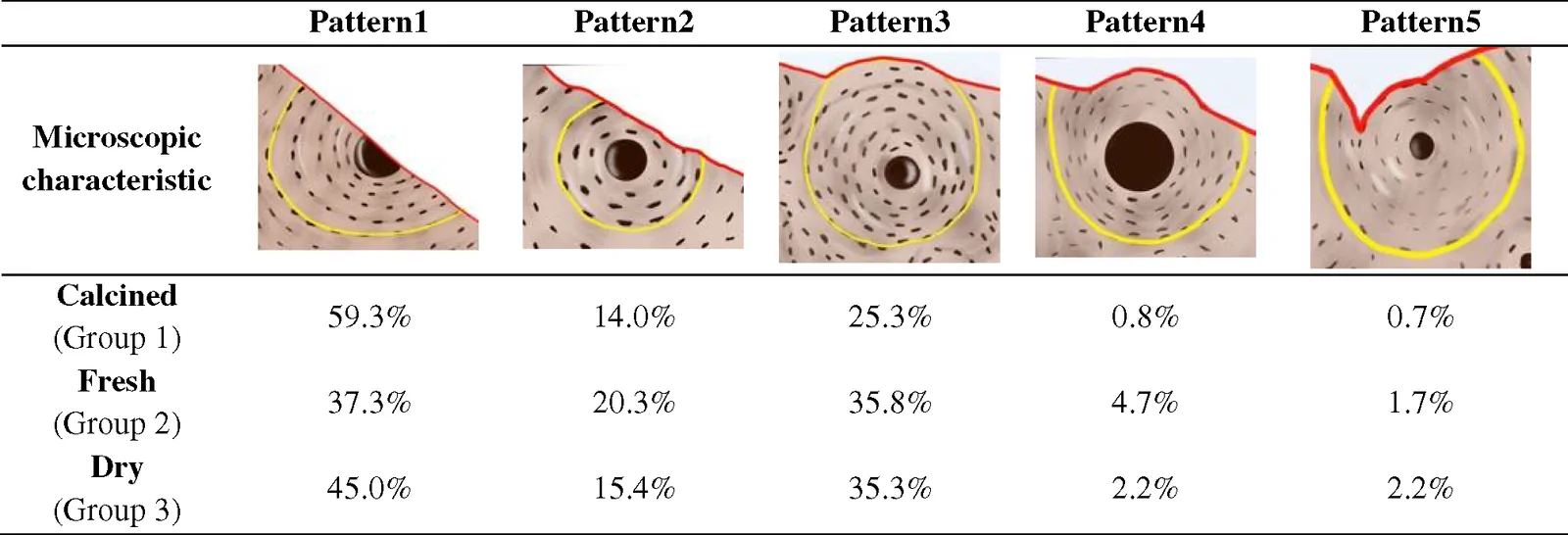
Summary of the different microscopic fracture characteristics detected in each group with related percentages along all sample considered

The associations between microscopic fracture characteristics and groups were also confirmed by the Chi-square analysis: Group 1 was enriched in Pattern 1, Group 2 in Patterns 2 and 4, while Group 3 showed no specific pattern preference. Furthermore, the cumulative frequencies of Patterns 1–2 and 3–4 in Groups 2 and 3 align with the A/B model proposed by Pechnikova et al. [20], from which this sample was derived. As for the calcined bones, despite their dehydration, heat-induced fractures behave similarly to those produced by high-velocity trauma, which, as noted by Piekarski [22], typically penetrate the Haversian canals. This phenomenon can be attributed to the fact that high-impact velocity prevents efficient dissipation of the applied force within the bone tissue, thereby reducing the bone’s elastic response time and exacerbating the extent of the damage [23]. Additionally, research by Sexton et al. [23] supports the hypothesis that distinct patterns of osteonal damage exist in human long bones subjected to blunt force trauma and gunshot wounds. Specifically, in cases of blunt force trauma, it is suggested that load-induced microfractures propagate from the Haversian canal toward the cement line, as osteocyte lacunae and Haversian canals act as stress concentrators [23]. Sexton et al. [23] reported that fractures analogous to Pattern 1—those crossing the Haversian canal—are more indicative of blunt force trauma than gunshot injuries. In line with these findings, both perimortem and postmortem samples in the present study, which were subjected to blunt force trauma, showed a high frequency of this pattern, despite the presence of additional osteonal damages. Notably, fractures crossing the Haversian canal were even more prevalent in the calcined group, suggesting a possible correlation between thermal alteration and this fracture pattern. This observation warrants further investigation into how different mechanical traumas and thermal exposure influence microscopic fracture characteristics. Moreover, the dominance of Pattern 1 in calcined bones with longitudinal fractures challenges previous assumptions—such as those by Schmidt and Symes [34], who associated Haversian canal-crossing fractures primarily with transverse breaks. Future research should explore the microstructural features of various fracture morphologies in calcined bones (e.g., longitudinal, transverse, step, conchoidal, burn line, patina, and delamination fractures) to assess whether osteonal fracture patterns vary with fracture type. Such analyses may not only enable intra-group characterization but also clarify whether certain patterns are more diagnostic in fracture types other than longitudinal.

Overall, although significant differences were observed among the three groups, no single pattern proved exclusive to a specific condition, despite some notable associations. This underscores the need for further research to better delineate the similarities and distinctions between heat-induced and mechanical trauma, and to identify microscopic features that may serve as reliable discriminators. Such investigations could not only enhance intra-group characterization but also determine whether certain fracture patterns are more diagnostic in non-longitudinal fracture types.

This research has several limitations that need to be considered. First, the analysis focused exclusively on longitudinal fractures –the most common type of heat-induced traumas– in diaphyseal segments of long bones. Additionally, the study was performed on a limited sample size because of the challenges in acquiring human remains and the lengthy procedure of thin-section preparation. The question is even more complicated by the fact that Europe still lacks a unified, clear and rigorous legal framework allowing the use of bodies donated to science for research and teaching [63]. However, future studies should perform similar analyses in larger human samples evaluating different types of fractures (e.g., conchoidal, transverse, step fractures, delamination) and bones (e.g., cranial bones) to allow more comprehensive results [4, 58]: the bone response to stresses, such as trauma and heat, varies according to the type of bone and its embryological origin [58], as well as to individual factors such as age [58] and sex [25], which were not considered in this study.

We emphasize the importance of conducting similar studies on human bone, as non-human samples present structural differences that warrant caution in interpretation—such as the frequent absence of secondary osteons and the presence of species-specific microstructural features (e.g., osteon banding, fibrolamellar bone) [64]. A key limitation of this study is that all calcined samples were obtained from a cremation oven, a highly controlled environment that limits the applicability of findings to less ideal scenarios, such as open-air or vehicle fires. Moreover, the uniformity of the cremation setting precluded stratification by exposure duration and temperature, hindering the analysis of thermal lesion progression under variable conditions [4]. Since both heating and cooling rates influence bone microstructure [25], future studies would benefit from experimental designs including a direct comparison between bones with exclusively heat-induced fractures and those with known perimortem mechanical injuries subsequently subjected to thermal exposure. Ideally, such experiments should involve soft-tissue-covered specimens to better simulate real forensic contexts. In this regard, Franceschetti et al. [11] demonstrated that perimortem fractures exhibit distinct macroscopic features—rounded margins and a brown, mottled, matte surface—that contrast with the sharp, glossy white appearance typical of heat-induced fractures. These macroscopic differences have been attributed to bleeding and clotting at perimortem fracture margins, which influence the bone’s response to heat and the coloration of blood residues, thereby contributing to the distinctive appearance of perimortem fractures. In this context, microscopic analysis may reveal blood-related alterations along the fracture margins, offering potential diagnostic markers to differentiate between heat-induced and trauma-related fractures. Regarding the methodology, this study employed optical microscopy exclusively to evaluate patterns of osteonal damage. Scanning Electron Microscopy (SEM) could serve as an additional analytical tool for this purpose. SEM has been widely used to examine macroscopic and ultrastructural changes in thermally altered bone, such as mineral recrystallization and organic matrix loss [27], as well as to evaluate sharp and ballistic injuries [60, 65, 66], but it has not been applied to investigate fracture behavior induced by fire and heat. Recent studies suggest that combining digital microscopy, 3D modeling, and Color Curvature Models (CCMs) can improve the interpretation of perimortem from heat-induced fractures at the macroscopic level [58], indicating that the microscopic level may provide additional, crucial insights. We are confident that, moving forward, the integration of diverse methodological approaches, including the comprehensive analysis of both macroscopic and microscopic features, will significantly advance forensic applications. Such a combined approach can provide innovative insights and improve the interpretation of trauma in the context of additional taphonomic changes, enhancing forensic investigative methods.

## Conclusions

This study investigated the microscopic fracture characteristics of longitudinal heat-related fractures, with a focus on osteonal damage patterns. Analysis of 1364 osteons from 99 thin sections, encompassing heat-induced, perimortem, and postmortem blunt force fractures, revealed that heat-related fractures tend to traverse the Haversian canal, whereas blunt force fractures either cross through osteons or follow their cement line. Despite significant variations in fracture pattern distributions, no pattern was exclusive to a specific group. The lack of a distinctive pattern in the analyzed samples prevents the development of a method for differentiating longitudinal thermal lesions from perimortem and postmortem mechanical injuries using optical microscopy. As such, this study serves not as a comprehensive investigation but as a call for further research into the microscopic analysis of thermal lesions.

Given the complexity of this issue, future studies expanding the human bone sample size and including a broader range of fracture types are needed to provide more conclusive data and a comprehensive understanding of heat-induced bone alterations.
